# Harnessing immunotherapeutic molecules and diagnostic biomarkers as human-derived adjuvants for MERS-CoV vaccine development

**DOI:** 10.3389/fimmu.2025.1538301

**Published:** 2025-03-13

**Authors:** Abdullah R. Alrasheed, Maaweya Awadalla, Hadeel Alnajran, Mohammed H. Alammash, Adil M. Almaqati, Ishtiaq Qadri, Bandar Alosaimi

**Affiliations:** ^1^ Department of Biological Sciences, Faculty of Science, King Abdulaziz University, Jeddah, Saudi Arabia; ^2^ Research Center, King Fahad Medical City, Riyadh Second Health Cluster, Riyadh, Saudi Arabia; ^3^ Department of Clinical Laboratory Sciences, College of Applied Medical Sciences, King Saud University, Riyadh, Saudi Arabia; ^4^ Riyadh Regional Laboratory, Ministry of Health, Riyadh, Saudi Arabia

**Keywords:** MERS-CoV, immunotherapeutic molecules, human-derived adjuvants, diagnostic biomarkers, vaccine development

## Abstract

The pandemic potential of the Middle East Respiratory Syndrome Coronavirus (MERS-CoV) highlights the critical need for effective vaccines due to its high fatality rate of around 36%. In this review, we identified a variety of immunotherapeutic molecules and diagnostic biomarkers that could be used in MERS vaccine development as human-derived adjuvants. We identified immune molecules that have been incorporated into standard clinical diagnostics such as CXCL10/IP10, CXCL8/IL-8, CCL5/RANTES, IL-6, and the complement proteins Ca3 and Ca5. Utilization of different human monoclonal antibodies in the treatment of MERS-CoV patients demonstrates promising outcomes in combatting MERS-CoV infections *in vivo*, such as hMS-1, 4C2H, 3B11-N, NBMS10-FC, HR2P-M2, SAB-301, M336, LCA60, REGN3051, REGN3048, MCA1, MERs-4, MERs-27, MERs-gd27, and MERs-gd33. Host-derived adjuvants such as CCL28, CCL27, RANTES, TCA3, and GM-CSF have shown significant improvements in immune responses, underscoring their potential to bolster both systemic and mucosal immunity. In conclusion, we believe that host-derived adjuvants like HBD-2, CD40L, and LL-37 offer significant advantages over synthetic options in vaccine development, underscoring the need for clinical trials to validate their efficacy.

## Graphical Abstract

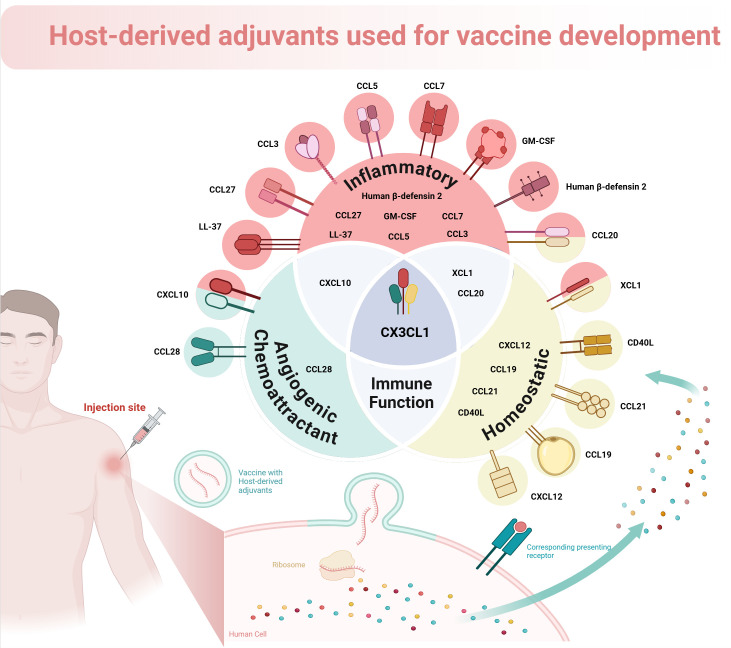

## Introduction

Middle East respiratory syndrome (MERS), a zoonotic disease caused by a member of the *Coronaviridae* family, was discovered in 2012 in Jeddah, Saudi Arabia (1, 2). This disease primarily targets the lower respiratory tract, eliciting host responses ranging from asymptomatic to severe acute respiratory syndrome, and may also impair other tissues, such as the kidneys (3, 4). Camels serve as the main reservoir for the virus and bats are considered the initial reservoir (5). Transmission to humans occurs through direct contact with infected camels or the consumption of their products (6). Between April 2012 and April 2024, the World Health Organization (WHO) recorded 2613 laboratory-confirmed cases from 27 countries, with approximately 36% (943 cases) resulting in mortality. Most of these cases - approximately 2204 occurrences with 862 deaths, representing a mortality rate of 39% - were documented in Saudi Arabia (7). Adults aged 50–59 exhibited the highest vulnerability to initial infection, whereas those aged 30–39 had the greatest risk for secondary infection (7). The case fatality rate (CFR) is highest among individuals aged 70–79 years, regardless of whether the infection was new or recurring (7).

The mean incubation period for MERS-CoV is approximately five days - although variations from 2–14 days occur (8, 9) - during which the host exhibits no symptoms of infection (9). Clinical manifestations of the illness vary widely, from mild symptoms such as cough, fever, and muscular discomfort, to severe conditions including pneumonitis, acute respiratory distress syndrome (ARDS), and respiratory failure (10). ARDS can result from cytokine release syndrome (CRS), which is characterized by an uncontrolled release of multiple proinflammatory cytokines due to an excessive immunological response by the host (11). To effectively understand the immunopathology of MERS-CoV, particularly MERS-CoV-induced CRS, acknowledgment of the potential overlap in the presentation and progression of severe MERS-CoV infections, as well as the lack of effective treatment options, is crucial.

COVID-19 pandemic has fast-forward the development of next generation vaccines. mRNA vaccines, like those developed by Pfizer-BioNTech and Moderna for COVID-19, use lipid nanoparticles to deliver genetic instructions for viral proteins, allowing for swift production and potent immune stimulation (12). Viral vector platforms, exemplified by AstraZeneca’s adenovirus-based vaccine, introduce genetic material to trigger immunity. Progress in structural vaccinology and nanoparticle engineering, as seen in Novavax’s SARS-CoV-2 vaccine, improves antigen presentation and durability (13). These innovations offer the potential for faster development, wider pathogen coverage, and enhanced thermostability, although expanding production and ensuring fair global distribution remain significant challenges. In contrast to SARS-CoV-2, MERS-CoV lacks approved preventive or therapeutic interventions, leaving supportive care as the only option. A vaccine could potentially curb transmission in high-risk regions, protect healthcare personnel, and mitigate pandemic risks associated with viral evolution or increased human-animal interactions. Moreover, lessons from COVID-19 emphasize the importance of proactive vaccine platforms against coronaviruses, which could be adapted for emerging variants.

This review aimed to explore the inflammatory biomarkers associated with MERS-CoV to ascertain whether MERS-CoV is linked to a unique inflammatory profile. A variety of immunotherapeutic molecules and diagnostic biomarkers that could be used in MERS vaccine development as human-derived adjuvants have been identified. The review also explores the possibility of identifying therapeutic agents and diagnostic markers targeting MERS-CoV, and contributes significantly to the fields of vaccinology and immunology by discussing the role of host-derived adjuvants in vaccine formulation.

## Diagnostic biomarkers

Addressing clinical MERS-CoV infections poses significant challenges, given the severity of the symptoms (14). Identifying a biomarker indicative of disease progression is crucial for diagnostic kit development. Cytokines and chemokine molecules can help to predict disease severity. The most prevalent cytokines and chemokines that could be diagnostic biomarkers for MERS (**Table 1**) are reviewed.

**Table 1:** Molecules that could be used as diagnostic markers.

Molecule	Function	Molecule role during infection	Treatment	Reference
IP10/CXCL10	Induces chemotaxis, proliferation, and inhibition of cell migration and proliferation	• Stimulating neutrophils in the lungs • Increases CXCL10 production • Releases oxidative burst via TLR4 •Induces lung inflammation, leading to ARDS	Antibodies targeting CXCL10	(17)
MCP-1/CCL2	Modulates monocyte circulation and infiltration enhances the production of memory T-cells and NK cells	• Th1 cells produce GM-CSF, stimulating monocyte and macrophage activation • Stimulation leads to maturation of CD14+ CD16+ monocytes • Monocytes migrate to the lungs, causing cytokine storm	Inhibiting MCP-1 activity	(23, 24)
CXCL8/IL-8	Influences neutrophil recruitment, activation, gathering, and NET initiation	• Leads to increased numbers of neutrophils in BAL fluid. • Releases myeloperoxidase and elastase • Potentially causes acute lung injury, pneumonia, and ARDS	Humanized anti-CXCL8 antibody	(30, 31)
CCL5	Attracts monocytes, T-cells, eosinophils, and is crucial for platelet activation and the coagulation cascade	• Increased neutrophil infiltration and production of MIP-2, IP10, and MCP-1 • Leads to lung damage and ARDS development	Met-RANTES therapy	(36–38)
IL-6	Induces monocyte regulation and macrophage development, modulates antigen-dependent B-cell differentiation, enhances B-cell IgG synthesis, and stimulates Th2 response by inhibiting Th1 polarization	• TNF, IL-1b, and IL-6 increase trypsin production breaking down matrix metalloproteinases and increasing tissue permeability • IFNγ production by Th1 cells is essential for antiviral immunity • IL-6 can reduce Th1 polarization by initiating CD4+ cell differentiation into Th2 cells or reducing IFNγ production • IL-6 promotes Th17 cell growth and IL-17A release, activating Bcl-XL • IL-17 increases neutrophil movement and viability, causing ARDS	Tocilizumab (IL-6 receptor inhibitor)	(55–59)
C5a and C3a	chemotactic for neutrophils, monocytes, eosinophils, and T-lymphocytes	• Promote phagocytic cell stimulation and synthesis of TNF-α, IL-1β, IL-6, and IL-8 • Enhance microvascular thrombosis, fibrinolysis, and vascular dysfunction • Elevated levels of C5a and C3a in the lung are linked to immune damage, disease severity, and ARDS development	eculizumab	(29, 74, 75, 79)

Interferon gamma-induced protein 10 (IP10/CXCL10) has been suggested as a biomarker for severe MERS-CoV infection. Kim et al. reported that CXCL10 levels were highest in patients during the second and third weeks of onset with severe MERS (13), compared with those with mild disease. Hong et al. indicated that CXCL10/IP10 concentrations were significantly elevated in patients who did not survive compared with those in surviving patients with MERS (15). Min et al. observed that patients who developed pneumonia during MERS infection exhibited high IP10/CXCL10 levels, which often decreased during the therapy phase in individuals who successfully recovered from pneumonia (16). The main role of CXCL10 are to mediate chemotaxis, and to inhibit cell migration and proliferation (17). CXCL10 plays a crucial function in stimulating migration, and infiltrating certain subsets of T lymphocytes at the infection sites during a viral infection (18). Elevated CXCL10 concentration has been associated with lung injury, as it promotes neutrophil infiltration into the lungs, leading to increased CXCL10 production and the release of oxidative bursts by neutrophils through Toll-like receptor 4 (TLR4) activation, resulting in ARDS (17). The role of this chemokine in viral infection can be protective or pathogenic, depending on host immunity and the type of virus (17). Considering its increased expression in previous research, CXCL10 appears to play a pathogenic role in MERS infection. Consequently, the development of antibodies targeting CXCL10 might offer a promising therapeutic strategy for treating ARDS, as demonstrated in the H1N1 mouse model of influenza A virus (19).

Monocyte chemoattractant protein-1 (MCP-1/CCL2) has been identified as a diagnostic marker for MERS-CoV progression. Alhetheel et al. reported that patients with symptomatic MERS who did not survive exhibited higher MCP-1 levels than those who recovered (2139 ± 548.2 vs. 776.5 ± 165.3 pg/mL; p < 0.004) (20). Furthermore, Hong et al. found that MCP-1 levels were significantly upregulated in patients with MERS who did not survive compared with levels in those who survived (15). Shin et al. demonstrated that plasma MCP-1 concentration was elevated fourfold in patients with severe and moderate disease (21). CCL2/MCP-1 modulates the circulation and infiltration of monocytes, memory T-lymphocytes, and natural killer (NK) cells, promoting inflammatory activities in tissues, particularly in the lungs (22). The upregulation of MCP-1 may activate T helper-1 (Th_1_) cell responses (23). Th1 cells produce granulocyte-macrophage colony-stimulating factor (GM-CSF), which may stimulate monocyte and macrophage activation. In individuals with coronavirus disease of 2019 (COVID-19), this stimulation leads to the maturation of CD14^+^ CD16^+^ monocytes, which release interleukin 6 (IL-6) (24). After migrating to the lungs, these monocytes exacerbate the cytokine storm, damaging the lungs (25). Therefore, inhibiting MCP-1 activity could be a therapeutic approach for treating MERS severity. Chirathaworn et al. demonstrated that MCP-1 is a potential biomarker implicated in immunopathological processes induced by Chikungunya virus, and is viewed as a possible therapeutic target (26). The severity of COVID-19 and potential mortality risk in patients can be predicted by biomarkers IP-10 and MCP-1, which serve as indicators of disease progression (22, 27). In addition, Tsaur et al. found that during the development of prostate cancer, chemokines undergo substantial alterations, with CCL2 emerging as a potential diagnostic indicator (28).

Chemokines such as CXCL8/IL-8 have been proposed as biomarkers for the severity of MERS infection. Patients with MERS-CoV who did not survive exhibited significantly higher levels of CXCL8 compared with those who survived (29). Alosaimi et al. demonstrated a significant correlation between the mortality rate of individuals with MERS-CoV and elevated levels of CXCL8 expression, compared to healthy controls (30). The chemokine CXCL8 influences key mechanisms, including neutrophil recruitment, activation, and aggregation, as well as the initiation of neutrophil extracellular traps (NETs) (30). Increased levels of CXCL8 leads to a higher concentration of neutrophils in the bronchoalveolar lavage (BAL) fluid, resulting in the release of myeloperoxidase and elastase. These compounds have the potential to cause acute lung injury, potentially progressing to pneumonia and ARDS (31). Additionally, CXCL8 enhances the production of CD4^+^ molecules and the activity of T helper cells during MERS infection (32). Consequently, humanized anti-CXCL8 antibody treatment has been shown to prevent lung neutrophil infiltration and alleviate acute lung injury syndrome, as demonstrated in rabbit models (33).

RANTES (CCL5) is another chemokine suggested as a diagnostic marker of the severity of MERS-CoV infection. Patients with MERS-CoV exhibited upregulated expression of CCL5, associated with disease severity (29). CCL5 effectively attracts monocytes, T-cells, and eosinophils (34). It is pivotal in activating platelets and initiating coagulation cascade (35). However, two different studies reported that CCL5 levels were significantly higher in recovered patients with MERS than in those with mild or severe disease (16, 21). The elevated RANTES levels may be linked to the release of this chemokine by activated virus-responsive T-cells (21). Elevated CCL5 levels in the lungs have been associated with increased neutrophil infiltration and the production of MIP-2, IP10, and MCP-1 in transgenic mice, leading to lung damage and ARDS development (36, 37). Additionally, CCL5 was elevated in RSV-infected and eosinophilic disease-sensitized mice. Met-RANTES therapy reduced inflammatory cell recruitment and local cytokine production (38).

CXCL10 and CXCL8 and CCL-5 are proinflammatory chemokines that play critical roles in the pathogenesis of infection, and function as prognostic indicators of coronaviruses severity (30, 39–43). CXCL10 is secreted by various cells, including monocytes, endothelial cells, and fibroblasts, in response to IFN-γ (44). CXCL8 is also secreted by numerous cell types in response to IL-6 and TNF-mediated cytokines, while antigen-presenting cells and activated T lymphocytes produce and release CCL5 (45–47). The concentration of CXCL10 in blood serum could serve as a potential indicator for identifying severe cases of *Mycoplasma pneumoniae* pneumonia in pediatric patients (48). CXCL10 has been found to be the most promising indicator for detecting acute Zika virus infection in potential clinical applications (49). CXCL10 and CXCL8 may serve as serum biomarkers for predicting liver injury induced by hepatitis B virus (HBV) infection (50). Gastric cancer progression can be predicted by using CXCL8 as a potential biological marker (51). Hu et al. found that concentrations of CCL5 in blood serum proved effective in distinguishing cirrhosis from chronic hepatitis B (CHB), with CCL5 emerging as the most dependable indicator (52). Moreover, CCL5 was initially recognized as an immunological and prognostic biomarker for cancer patients (53).

Interleukin-6 (IL-6) could help to predict disease progression in MERS-CoV-infected patients. Kim et al. revealed a significant increase in IL-6 levels in patients with severe MERS up to the third week after symptom onset (54). In another study, plasma IL-6 concentration was considerably elevated and was correlated with MERS infection severity (21). Hong et al. showed that IL-6 levels were highly upregulated in patients who did not survive compared to those who survived (15). IL-6 regulates multiple immune-stimulating pathways, which in turn influence the host defense. These pathways include: the regulation of monocytes and their development into macrophages, modulation of antigen-dependent B-cell differentiation, enhanced IgG synthesis by B-cells, and stimulation of Th2 response via Th1 polarization inhibition (55). IL-6 levels have been shown to be associated with the severity of lung inflammation in a study of influenza virus (56). IFNγ produced by Th1 cells is crucial for a successful antiviral immune response. IL-6 hinders Th1 polarization via the stimulation of CD4^+^ cells to transform into Th2 cells or by decreasing IFNγ production (57). IL-6 also enhances Th17 development and stimulates the release of IL-17A, which in turn activates antiapoptotic molecules such as Bcl-XL. This supports the survival of cells that have been infected by a virus (58). Simultaneously, IL-17 enhances the movement and viability of neutrophils, which are involved in the development of ARDS in patients with COVID-19 (55, 59). Hence, treating patients who have increased IL-6 levels with tocilizumab, an IL-6 receptor inhibitor, could be effective against severe MERS cases, and has also provided therapeutic advantages in treating COVID-19 (60, 61). This treatment is now considered one of the most promising options available (62).

IL-6, a proinflammatory cytokine, has been found to have increased expression in various conditions, including respiratory ailments, cancer and viral infections, such as HIV and HCV. Significantly elevated levels of IL-6 have been observed in patients with severe cases of severe acute respiratory syndrome (SARS), MERS, and COVID-19 compared to milder cases (15, 54, 63–71) and is considered as an indicator for MERS progression. Santa Cruz A et al. demonstrated that IL-6 serves as a valuable instrument for assessing prognosis, particularly in predicting patient outcomes (72). In addition, IL-6 has been recommended to be a diagnostic biomarker for gastric cancer (73).

Complement anaphylatoxins, such as C5a and C3a, can be used as markers for predicting the progression of MERS-CoV infection. Hamed et al. revealed that MERS-CoV-infected patients had elevated levels of C5a and C3a, which were positively associated with severity and mortality rates (29). C5a is a chemotactic agent for neutrophils, monocytes, eosinophils, and T-lymphocytes (74). Complement anaphylatoxins C3a and C5a are formed, following the overactivation of the pulmonary and systemic complement systems, in turn causing inflammation, endothelial cell damage, thrombus formation, intravascular coagulation, and, ultimately, death due to multiple organ failure (74–76). Following infection, complement anaphylatoxins promote the stimulation of phagocytic cells and the synthesis of TNF-α, IL‐1β, IL‐6, IL‐8, granular enzymes, and free radicals. These substances enhance the development of microvascular thrombosis, fibrinolysis, and vascular dysfunction (75–78). Elevated levels of C5a and C3a in the lung have been suggested in contributing to immune-related damage, disease severity, ARDS development, and higher mortality rates in MERS-CoV-infected patients (29). Patients with high levels of complement anaphylatoxins could be treated therefore with eculizumab, which is a human monoclonal antibody (hmAb) with a significant affinity for the complement protein C5 (79). This antibody blocks the separation of C5a and C5b and stops the production of the cell-destroying C5b-9 complement complex (80). Inhibiting the C5a-C5aR pathway in MERS-CoV infected hDPP4 transgenic mice led to a decrease in the extent of infection-induced tissue damage (81). Patients with COVID-19 demonstrated a rapid, significant, and evident response to eculizumab, resulting in complete recovery, despite severe lung injury (79).

Complement proteins C3a and C5a have been found to be biomarkers of MERS and COVID-19 severity. C5a serves as a potent chemoattractant, facilitating the recruitment of inflammatory cells (neutrophils, eosinophils, monocytes, and T lymphocytes), induces the activation of phagocytic cells, and elicits the release of granule enzymes and oxidants (82). C3a effectively activates eosinophils, inducing granule release, reactive oxygen intermediate generation, and chemotaxis in *in-vitro* assays (83). A study by Alosaimi et al. demonstrated that C5a and C3a can be prognostic biomarkers of COVID 19 severity (84). In addition, C5a has been considered to be a potential marker of severity in patients with myasthenia gravis (85). C3a could serve as an indicator for early identification of hepatitis C virus-associated hepatocellular carcinoma (86).

## Human immunotherapeutic molecules

Currently, MERS is the most fatal human coronavirus-related disease, with a mortality rate exceeding 35% (14, 62), with no verified antiviral treatments available. Identifying markers that enhance the effectiveness of treatment is crucial. Our study investigates the most common human derived molecules that could aid in treating MERS (**Table 2**).

**Table 2:** Molecules that could be used for treatment.

Molecule	Number of patients/Animal model	Type of study	Result	Reference
IFN-α2a or IFN-β1a and ribavirin	32	Retrospective study	No efficacy of these combinations	(91)
IFN-α2a and ribavirin	20	Retrospective cohort study	An increased survival rate within 14 d but not within 28 d	(92)
Recombinant IFN-α2a, IFN-β1a, or IFN-α2b and ribavirin	144	Retrospective cohort study	Did not reduce the mortality rate within 90 d	(93)
Mersmab-1 (hMS-1)	hDPP4-Tg mice	*In vivo*	Complete protection against lethal MERS-CoV infection	(98)
4C2h	Ad5-hCD26-transduced mice	*In vivo*	Lowered MERS-CoV viral concentrations in the lung tissue	(99)
3B11-N	Rhesus monkey	*In vivo*	Markedly decreased pulmonary damage	(100)
NbMS10-Fc	hDPP-4 Tg mice	*In vivo*	completely protecting humanized mice from lethal MERS-CoV infection	(101)
HR2P-M2	Ad5-hCD26-transduced mice	*In vivo*	A reduction in viral titer	(102)
HR2P-M2 + INF-β			An additional reduction in infection.	
SAB-301	Ad5-hDPP4-transduced mice	*In vivo*	Rapidly reduced viral lung titers	(103)
M336	Rabbit	*In vivo*	Decreased MERS-CoV levels in rabbit lungs	(106)
	hDPP4-Tg mice	*In vivo*	Complete preventive and curative protection	(107)
	Common marmoset	*In vivo*	Mitigated the disease’s intensity and failed to provide total protection against MERS-CoV	(108)
LCA60	Ad5-hDPP4 mice	*In vivo*	Reduction of MERS-CoV titer in the lungs	(109)
REGN3051 and REGN3048	huDPP4 mice	*In vivo*	Exhibited efficacy in reducing MERS-CoV replication	(111)
MCA1	common marmosets	*In vivo*	Effectively suppressed the replication of MERS-CoV	(113)
MERS-GD27	hDPP4-Tg mice	*In vivo*	Decreased viral loads (both infectious virus and viral RNA) within the pulmonary tissue	(116)

Interferon (IFN) has been used for viral treatment. Type I interferon (IFN-I) is the first cytokine upregulated after infection, activating approximately 300 genes involved in immunomodulation and antiviral defense (87, 88). Falzarano et al. demonstrated that administering IFN-α2b and ribavirin within 8 hours of viral exposure effectively reduced lung damage and decreased viral load in the lungs (89). However, this combination treatment provided no benefit when administered to severely ill patients with multiple comorbidities (90). A retrospective study involving 32 patients revealed no efficacy in treating MERS with IFN-α2a or IFN-β1a combined with ribavirin (91). In another retrospective cohort study, 20 patients with severe MERS-CoV infection were treated with IFN-α2a and ribavirin; this resulted in an increased survival rate within 14 days but not within 28 days (92). Arabi et al. conducted a retrospective cohort study involving 144 critical patients with MERS and treated with recombinant IFN-α2a, IFN-β1a, or IFN-α2b and ribavirin; however, no reduction was observed in the 90-day mortality rate (93). These combinations may be more effective in the early stages of the disease. Additionally, marmosets infected with MERS and treated with IFN-β1b exhibited less severe illness and lower than average viral loads in the lungs and extrapulmonary organs during necropsy compared with those in untreated animals (94). INF-I used on SARS patients showed no effective results. A study by Wu et al. demonstrated that INF-α could potentially help reduce the duration of the clinical course (95). Loutfy et al. revealed that the combination of interferon alfacon-1 and corticosteroids was linked to several positive outcomes: a decrease in oxygen saturation impairment caused by the disease, faster improvement of lung abnormalities visible on radiographs, and reduced levels of creatine kinase (96). However, Zhao et al. found that administering both interferon and high doses of immunoglobulins yielded no significant results in combatting SARS infection (97).

Human immunotherapeutic agents have been tested against MERS-CoV infection. Mersmab-1 (hMS-1) is a neutralizing monoclonal antibody that specifically targets the MERS-CoV receptor-binding domain (RBD) with strong affinity. A study by Qiu et al. concluded that a single administration of hMS-1 effectively impeded MERS-CoV RBD from binding to its viral receptor. This intervention offered complete protection against lethal MERS-CoV infection in genetically modified mice that expressed human dipeptidyl peptidase 4 (hDPP4-Tg) (98).

A neutralizing monoclonal antibody named 4C2h was developed to target the receptor binding domain of MERS spike protein and inhibit viral entry. In their study, Li et al. showed that 4C2h effectively lowered MERS-CoV viral concentrations in the lung tissue of mice that were genetically modified with Ad5-hCD26 and later infected (99).

3B11-N, a human anti-MERS monoclonal antibody, has been tested against MERS-CoV infection *in vivo*. 3B11-N did not show any escape mutants during the initial characterization, demonstrated the highest virus neutralization ability, and was determined to be suitable for mass production, potentially providing significant therapeutic advantages (100). Johnson et al. illustrated that MERS-infected rhesus monkeys treated with 3B11-N exhibited markedly decreased pulmonary damage compared to infected individuals who received no treatment, suggesting that this antibody could be an effective therapy for MERS-CoV infection (100).

NbMS10-Fc, a neutralizing nanobody and its human-Fc-fused version, is a protective treatment against MERS-CoV. NbMS10 exhibited strong binding affinity to the MERS-CoV RBD and inhibited interaction between RBD and DPP4 (101). A study by Zhao, et al. showed that administering a single dose of NbMS10-Fc exhibited exceptional prophylactic and therapeutic efficacy, completely protecting humanized mice from lethal MERS-CoV infection (101).

The peptide MERS-CoV fusion inhibitor HR2P-M2, which specifically targets the S protein HR1 domain, demonstrates significant efficacy in suppressing both *in vitro* and *in vivo* infections caused by various strains of MERS-CoV (102). Intranasal administration of HR2P-M2 protected mice expressing human dipeptidyl peptidase 4 via adenovirus serotype-5 from MERS-CoV infection, and was effective against viral strains with and without HR1 region mutations in the S protein (102). The protective effect was enhanced when combined with INF-β, which indicates promising prospects for its advancement as a preventive measure, and highlights its potential application as a treatment option for patients infected with MERS-CoV (102).

SAB-301 is a trans-chromosomic human IgG immunoglobulin (Tc hIgG), derived from purified Al-Hasa strain MERS-CoV spike protein nanoparticles. Single doses of SAB-301 administered to Ad5-hDPP4 receptor–transduced mice before or after MERS-CoV infection rapidly reduced viral lung titers (103). A clinical trial, registered with ClinicalTrials.gov (number NCT02788188), was conducted to evaluate SAB-301 safety and tolerability. It indicated that SAB-301 exhibits safety and tolerability at 50 mg/kg, which may be therapeutically effective (104).

M336 are human monoclonal antibodies that target the RBD of the MERS-CoV spike glycoprotein and interact with CD26/DPP4 (105). Research conducted *in vivo* revealed that preventive treatment with m336 decreased MERS-CoV levels in rabbit lungs (106). M336 also offered complete preventive and curative protection against MERS-CoV in genetically modified mice expressing human DPP4 (107). However, A separate investigation involving a non-human primate - the common marmoset - indicated that m336 only mitigated the disease’s intensity and failed to provide total protection against MERS-CoV (108).

LCA60 is an additional human neutralizing monoclonal antibody developed to combat MERS-CoV. This antibody was generated by isolating IgG memory B cells from an individual infected with MERS and then immortalizing these cells through the use of the Epstein-Barr virus (109). The antibody LCA60 demonstrates efficacy in neutralizing MERS-CoV infection in cellular models and offers both preventive and therapeutic protection in BALB/c mice that have been modified with adenoviral vectors to express hDPP4 (110). In a more challenging model using IFN-α/β receptor-deficient mice expressing hDPP4, LCA60 treatment led to a substantial decrease in viral load within the lungs (109). This reduction occurred more rapidly compared to BALB/c mice, with a three-log decrease observed in just one day, as opposed to the three days required in BALB/c mice (109).

Other human neutralizing monoclonal antibodies were developed to protect and treat MERS-CoV infection: REGN3051 and REGN3048. REGN3051 and REGN3048 were produced by immunizing humanized transgenic mice (VelocImmune mice) with DNA encoding the MERS-CoV S protein to engineer hybridoma B cells that produce neutralizing monoclonal antibodies (111). A study by Pascal et al. conducted *in vivo* revealed that REGN3051 and REGN3048 inhibited MERS-CoV multiplication in mice with humanized DPP4, both as a preventive measure and as a treatment (111). However, when tested in common marmosets, these monoclonal antibodies appeared to be more efficient in preventing MERS-CoV infection, rather than treating it once established (112).

A human monoclonal antibody, MCA1, was identified by isolating B cells from a patient who had previously overcome MERS, targeting the receptor-binding domain of the MERS-CoV S glycoprotein (113). MCA1 demonstrated strong neutralizing activity against MERS-CoV in cell entry assessments. *In vivo*, MCA1 effectively suppressed the replication of MERS-CoV in common marmosets when given as a preventive or therapeutic treatment (113).

Two strong human neutralizing monoclonal antibodies, MERS-4 and MERS-27, were tested against MERS-CoV infection. MERS-4 and MERS-27 were derived from a non-immune human yeast display antibody library generated using polyadenylated RNA sourced from the spleen and lymph nodes of regular individuals (114). Both MERS-4 and MERS-27 effectively inhibited pseudovirus and live MERS-CoV from entering cells. The combined use of MERS-4 and MERS-27 demonstrated a synergistic effect on pseudotyped MERS-CoV. The primary approach to neutralizing MERS-4 and MERS-27 is by inhibiting the attachment of the RBD to DPP4 (114).

MERS-GD27 and MERS-GD33 are human neutralizing monoclonal antibodies that are produced from the whole blood of a MERS patient (115). MERS-GD27 and MERS-GD33 demonstrated the most potent neutralizing activity against pseudotyped and live MERS-CoV *in vitro*. Analysis of mutagenesis showed that MERS-GD27 and MERS-GD33 focused on distinct areas in the S glycoproteins. The synergy of the two monoclonal antibodies effectively neutralized pseudotyped MERS-CoV (115). A study conducted *in vivo* highlighted the prophylactic and therapeutic advantages of MERS-GD27 in protecting HDPP4-transgenic mice against MERS-CoV infection (116).

The neutralizing monoclonal antibody S309, isolated from the peripheral blood mononuclear cells of a patient infected with SARS-CoV in 2003, was tested against MERS-CoV infection. S309 demonstrated strong binding affinity for both SARS-CoV and SARS-CoV-2 (117). *In vivo*, the monoclonal antibody CR9114 exhibits neutralizing capabilities against both influenza A and B types, and CR6261 has shown the ability to lower mortality rates in mice infected with H1N1 and H5N1 influenza A subtypes (118, 119). Additionally, monoclonal antibodies 70-1F02 and 9-3A01 have demonstrated the capacity to inhibit infections caused by two H1N1/H1N5 influenza A subtypes (120, 121).

## Host-derived adjuvants in vaccine development

Inducing a robust memory response from T- and B-cells targeted toward the specific pathogen, along with the presence of durable plasma cells, is the primary objective of an effective immunization strategy against infectious diseases. The unavailability of vaccines specifically targeting MERS-CoV highlights the urgent need for targeted immune responses against the virus. Various strategies have been employed to develop a MERS-CoV vaccine. This study reviews the strategies used to generate human-derived vaccine adjuvants (**Table 3**).

**Table 3:** Molecules that could be used as vaccine adjuvants.

Molecule	Targeted betacoronavirus	Vaccine Substances	Animal Model	Adjuvant Effects	Reference
Human β-defensin 2	MERS	spike protein receptor-binding domain (S RBD) and HBD 2	hDPP4-Tg mice	Triggered strong adaptive immune responses targeting the specific antigen (Ag) and provided protection against MERS-CoV infection	(122)
CD40L	MERS	rAd5-S1/F/CD40L	hDPP4-Tg mice	Decreased pulmonary viral load	(130)
LL-37	MERS	S-RBD and LL-37	hDPP4-Tg mice and C57BL/6	Production of mucosal IgA and systemic IgG antibodies	(135)
CCL27	SARS-CoV-2	pCTACK; CCL27	Mice	Increased frequencies of interferon gamma (IFNγ)+ CD8+ T cells	(142)
GM-CSF	SARS-CoV-2	RBD plus pGM-CSF	Mice	induced CD4+ and CD8+	(141)

Human β-defensin-2 (HBD-2) has been used as a vaccine adjuvant against MERS-CoV. Human β-defensins (HBDs) are short host defense peptides produced by epithelial cells to create mucosal barriers that protect against different types of infectious agents (122). HBDs play a crucial role in connecting the activation of pathogen-specific innate and adaptive immunity by recruiting and activating different types of leukocytes such as macrophages, dendritic cells (DCs), and T cells (123–125). Kim et al. demonstrated that immunization of hDPP4-Tg with a fusion of spike protein receptor-binding domain S RBD and HBD 2 (S RBD-HBD 2) induced robust antigen-specific adaptive immune responses and conferred protection against MERS-CoV infection. Additionally, S RBD-HBD 2 immunization reduced the progression of pulmonary fibrosis in the lungs of MERS-CoV-infected hDPP4-Tg mice and suppressed the activation of endoplasmic reticulum stress signaling following viral infection (122).

Another human derived molecule that can be utilized as vaccine adjuvant for MERS-CoV is CD40L. CD40L, a membrane protein of type II, serves a critical function as a co-stimulatory molecule and essential regulator of immune function (126). The primary expression occurs temporarily on activated CD4+ T cells (127). The interaction between CD40L and its receptor CD40, found on all antigen-presenting cells (APCs), plays a crucial role in connecting innate and adaptive immune responses (128, 129). Research conducted by Hashem et al. demonstrated that hDPP4-Tg mice inoculated with a combination of non-replicating recombinant adenovirus 5 (rAd5), MERS-CoV S1 protein, and murine CD40L (rAd5-S1/F/CD40L), provided complete protection against MERS-CoV, as demonstrated by the significantly decreased pulmonary viral load (130).

LL-37, a human antimicrobial peptide, exhibits chemotactic properties and modulate the activities of various immune cells, including dendritic cells (131). During infection, LL-37 functions as an alarm signal, linking the innate and adaptive immune systems by attracting immune cells to the infection site (132). LL-37 has the potential to exhibit antiviral activity and regulate the delicate balance between pro- and anti-inflammatory responses by modulating inflammatory cytokine expression; therefore, these peptides may serve as effective vaccine adjuvants (133, 134). In their study, Kim et al. found that immunized mice with a combination of S-RBD and LL-37 (S-RBD-LL-37) stimulated the production of mucosal IgA and systemic IgG antibodies, which demonstrated virus-neutralizing capabilities (135).

**Figure 1:**
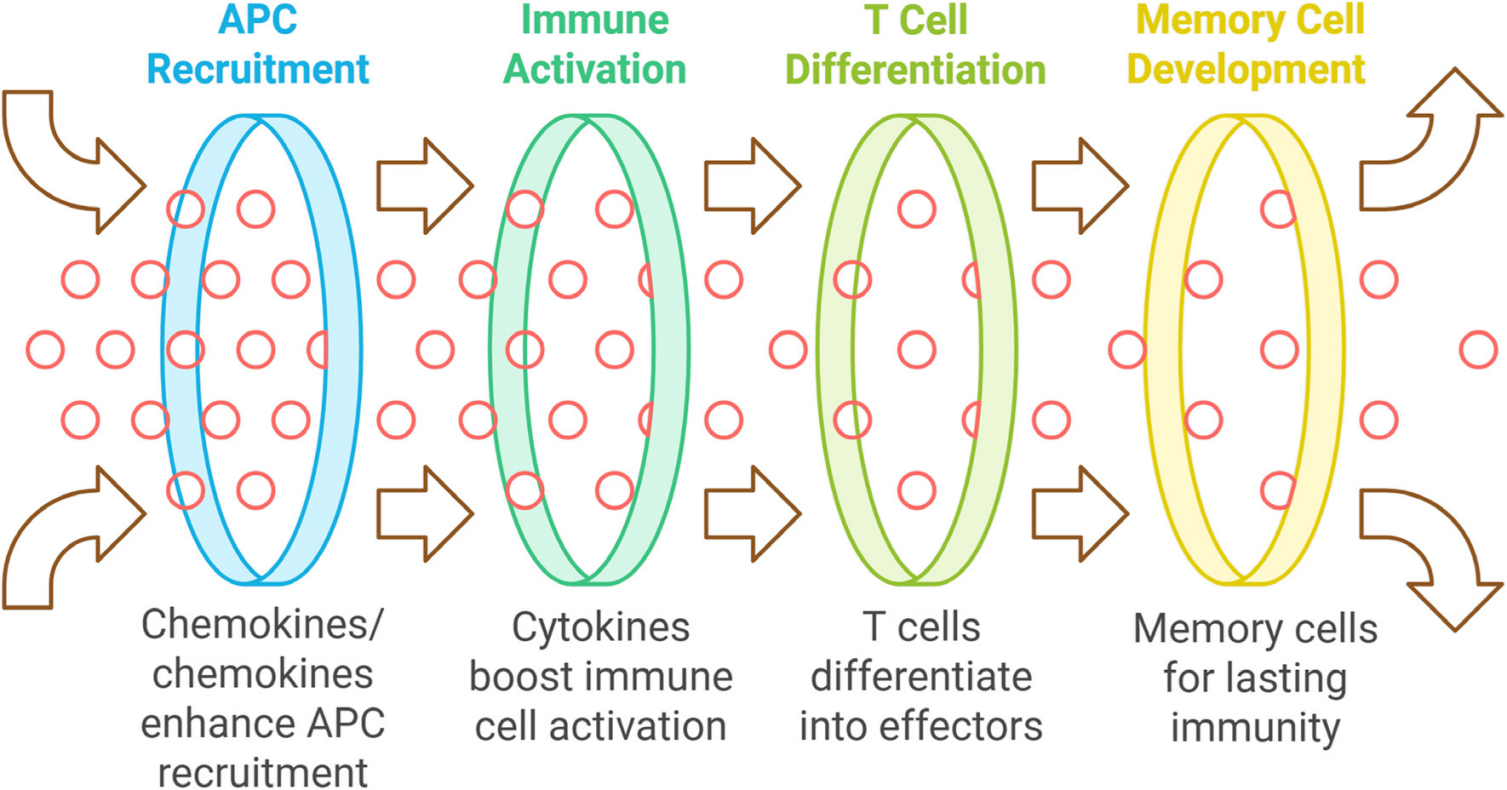
The mechanism of enhancing vaccine efficacy using host-derived adjuvants.

Chemokines enhance the recruitment of antigen-presenting cells (APCs) to vaccination sites, improving antigen uptake and T cell presentation, which is vital for a strong adaptive immune response (136–138). Cytokines directly boost immune cell activation and proliferation, aiding the differentiation of naive T cells into effector T cells necessary for infection clearance. They also help to develop memory T and B cells for lasting immunity post-vaccination (139) (see **Figure 1**). Host-derived cytokines and chemokines are generally better tolerated than synthetic adjuvants, which can trigger adverse immune reactions. Using the body’s own signaling molecules can optimize immune responses, and these substances are versatile for various vaccine types, including protein subunit, DNA, and viral vector vaccines (138).

Host-derived cytokines and chemokines are being explored as vaccine adjuvants to enhance immune responses by utilizing the body’s own signaling molecules. These proteins can modulate immune responses, potentially offering a safer and more effective alternative to synthetic adjuvants. Various cytokines, including interleukins and interferons, have shown promise in promoting antigen-specific immune responses when used with vaccines. Notable examples include CXCL10, CXCL12, CCL19, CCL5, CCL3, CX3CL1, IL-1, and INF-α, which have been tested in both murine and human studies. The selection of these adjuvants can either be homeostatic or inflammatory, influencing their effects on immunity (136, 139).

Previous studies have highlighted the effectiveness of cytokines like CCL28, GM-CSF, IL-2, IL-12, IL-15, IL-21, and IL-33 in enhancing immune responses to various vaccines (140, 141). Innovative approaches using host-derived cytokines have demonstrated improved systemic and mucosal immunity post-vaccination. For instance, the mucosal chemokine pCTACK (CCL27) has been shown to enhance vaccine responses to SARS-CoV-2, while GM-CSF (pGM-CSF) has been effective in DNA vaccinations against the virus by promoting antigen expression and immune cell recruitment (140–143).

## Human derived adjuvants used in clinical and pre-clinical studies

Adjuvants derived from human sources such as immune cells, cytokines, and proteins improve the efficacy of viral vaccines by boosting innate immunity, increasing antigen presentation, and enhancing overall immune responses. These adjuvants are primarily being evaluated for safety, efficiency and widespread applicability in clinical and preclinical research. Current research on notable human-derived adjuvants includes immuno-stimulating complexes (ISCOMs), aluminium salts, granulocyte-macrophage colony-stimulating factor (GM-CSF), interleukins (IL-12, IL-15), exosomes, dendritic cells (DCs), and monoclonal antibodies (mAb). Furthermore, Toll-like receptor (TLR) agonists (e.g., Imiquimod, CpG 7909) and MF59 (an oil-in-water emulsion) exhibit the potential to augment responses to vaccines (**Table 4**).

**Table 4:** Human derived adjuvants used in clinical and pre-clinical studies.

Adjuvant	Vaccine	Viral Target	Study Stage	Reference
ASO4 (Aluminium salt and MPL)	Cervarix	HPV types 16 and 18	Phase III clinical trials	(152, 186)
ASO4C (Aluminium phosphate and MPL)	Fendrix	HBV	Complete	(154)
ISCOMs	Influenza vaccine	Influenza virus	Pre-clinical	(187, 188)
ISCOMs	HSV vaccine	HSV	Pre-clinical	(189)
ISCOMs	RSV vaccine	RSV	Pre-clinical	(148, 190)
ISCOMs	Hepatitis B vaccine	HBV	Pre-clinical	(191)
IL-12	HIV Mag DNA vaccine	HIV	Phase I clinical trials	(166)
IL-15	DNA vaccine	HIV	Phase I clinical trials	(166)
Exosomes	Gp120-Texo/Gag-Texo	HIV	Preclinical	(177)
Exosomes	Hepatitis B vaccine	HBV	Preclinical	(177)
Exosomes	influenza vaccine	Influenza virus	Preclinical	(177, 192)
TLR7 Agonist (Imiquimod)	influenza vaccine	Influenza virus	Phase I clinical trials	(179)
TLR7 Agonist (Imiquimod)	HSV vaccine	HSV	Preclinical	(180)
GM-CSF	DNA vaccine against HIV-1 Gag	HIV	Preclinical	(193)
GM-CSF	Influenza vaccine	Influenza virus	Preclinical	(169–171)
TLR9 agonist CpG 7909	Engerix-B Vaccine	HBV	Phase I/II clinical trials	(181, 183)
TLR9 agonist CpG 7909	HEPLISAV^™^ hepatitis B vaccine	HBV	Phase III clinical trials	(182)
TLR9 agonist CpG 7909	HIV vaccine	HIV	Complete	(182, 183)
Dendritic cells	HIV Vaccine	HIV	Phase II clinical trials	(156)
Monoclonal antibodies	HBV Vaccine	HBV	Phase I clinical trials	(184)
MF59	FLUAD	Influenza virus	Clinical	(185)

ISCOMs, consisting of saponin, phospholipids, cholesterol, and antigens such as Quil A (144), serve as strong adjuvants for hydrophobic antigens, particularly those derived from enveloped viruses (145). The saponin-cholesterol matrix reduces toxicity and hemolytic activity (144), demonstrating robust cellular and humoral responses in both animal and human trials (144, 146). ISCOMs also induce strong mucosal and systemic immunity (147), rendering them interesting candidates for nasal vaccinations, including those for influenza (148).

Monophosphoryl lipid A (MPL), derived from salmonella minnesota in detoxified form, stimulates TLR4 on DCs, thereby augmenting innate immunity (149) and priming CD4+ and CD8+ T-cell responses to establish adaptive immunity (150) and immunological memory (151). MPL is utilized in Adjuvant System 4 (AS04) with aluminium salt, in HPV (Cervarix™) (152, 153) and HBV (Fendrix) vaccinations (154, 155). Published clinical trials indicate that DC immunotherapy in HIV-1 infection can provoke HIV-specific immune responses (156).

Type I interferons (IFNs) facilitate the maturation of DCs, hence augmenting the formation of antigen-specific CD8+ T lymphocytes for tumor suppression. Employing IFNs as adjuvants to vaccination may represent a promising strategy. IFNs possess a brief half-life but albumin conjugated to a protein will extend the half-life of the associated protein (157).

Cytokine adjuvants like IL-12 and IL-15 (158–160) boost immune responses. IL-12, produced by DCs and monocytes, is crucial for cellular immunity (161), where defects increase susceptibility to intracellular pathogens (162, 163). IL-15, produced by DCs, monocytes and epithelial cells, supports proliferation of B and T cells, activation of NK cells, and long-term memory cell responses (164, 165). Early clinical trials combining IL-12 or IL-15 with an HIV DNA vaccine show their potential as adjuvants (166).

GM-CSF improves vaccine effectiveness by stimulating DCs. GM-CSF genes (codon optimized) enhance protein expression and immunological responses, particularly against HIV-1 Gag (167). GM-CSF produces enhanced antibody responses to influenza vaccines (168) and demonstrates potential as an effective adjuvant in clinical trials (169–171).

Exosomes originating from infected cells can transmit viral components to adjacent cells, thereby eliciting antiviral immunity (172). The evolutionary parallels between viruses and exosomes indicate that exosomes may serve as viable vaccine platforms (173). Exosome-based HIV vaccines, such as Gag-Texo and Gp120-Texo, have demonstrated robust, tailored immune responses (174). Moreover, modified Nefmut-exosomes proficiently stimulate CTL responses against HIV and other viruses, including Ebola, HBV, and influenza (175, 176). Preliminary research indicates that exosomes may serve as adjuvants for influenza and HBV vaccines, augmenting immune responses and protection, hence reinforcing their potential as effective vaccine adjuvants (177).

TLR7 agonist Imiquimod augments vaccine immunogenicity by facilitating DC maturation and eliciting a Th1 response (178). Research in humans and animals demonstrates that it enhances and extends immune responses, especially in influenza and HSV vaccinations, affirming its efficacy as an adjuvant (179, 180). Similarly, TLR9 agonist CpG oligodeoxynucleotides (ODNs) stimulate plasmacytoid DCs and B cells, promoting Th1 and proinflammatory responses. As adjuvants, they augment antigen-presenting cell function, thereby fortifying humoral and cellular immunity. Preclinical and clinical experiments demonstrate that CpG ODNs enhance the efficiency of HIV and HBV vaccines (181, 182), with CpG 7909 being effective for immunocompromised patients (182, 183).

Creating HBV-specific neutralizing mAbs may facilitate the elimination of surplus viral proteins, perhaps reinstating adaptive immunity and augmenting the efficacy of antiviral medications. Fully human mAbs from individuals vaccinated against HBV and those who have recovered demonstrate potential as adjunctive therapies to diminish viral protein levels and enhance immunological recovery, hence improving the results of antiviral treatments (184). Immunosenescence results in diminished antibody responses to inactivated influenza vaccine (IIV) in elderly persons. To resolve this, adjuvants such as MF59, an oil-in-water emulsion, have been included to improve vaccine efficacy. Since 1997, MF59-adjuvanted IIV3 (FLUAD) has been authorized for older patients in Europe and exhibits superior immunogenicity compared to nonadjuvanted IIV, underscoring its significance in enhancing vaccine responses in the elderly (185).

## Conclusion

The Middle East Respiratory Syndrome Coronavirus (MERS-CoV) remains a significant global health threat. This review emphasized critical biomarkers linked to MERS-CoV infection. These biomarkers could improve clinical diagnostics, therapeutic interventions and vaccine development for MERS-CoV. The benefits of using host-derived adjuvants in vaccine development were also highlighted, focusing on their safety and effectiveness in enhancing immune responses. Disease progression of MERS-CoV can be estimated by assessing the levels of certain molecules, including CXCL10/IP10, CXCL8/IL-8, CCL5/RANTES, IL-6, and the complement proteins Ca3 and Ca5. However, further studies must be conducted to measure the level of cytokines and chemokines at different time points during the infection. Despite investigations into several therapeutic agents, such as interferons, their efficacy has proven inadequate. *In vivo* studies, various human monoclonal antibodies showed substantial benefits in fighting MERS-CoV infection. The antibodies tested include hMS-1, 4C2h, 3B11-N, NbMS10-Fc, HR2P-M2, SAB-301, M336, LCA60, REGN3051, REGN3048, MCA1, MERS-4, MERS-27, MERS-GD27, and MERS-GD33. This highlights the urgent need for ongoing clinical trials to discover more effective treatment options. Additionally, exploring vaccine adjuvants is crucial for advancing immunization strategies against MERS-CoV. MERS infections may be prevented by designing a vaccine containing human-derived molecules that includes one or more adjuvants, such as HBD-2, CD40L and LL-37. The potential of host-derived adjuvants, particularly cytokines and chemokines, offers a promising direction for enhancing vaccine effectiveness. These natural signaling molecules not only improve the recruitment of antigen-presenting cells (APCs) to vaccination sites but also promote robust activation and differentiation of T cells. By harnessing the body’s own immune mediators, adaptive immune responses can be optimized while minimizing the adverse effects commonly associated with synthetic adjuvants.

Evidence from both murine and human studies supports the use of various cytokines, including interleukins and interferons, as effective adjuvants that enhance antigen-specific immunity across diverse vaccine platforms, including protein subunit, DNA, and viral vector vaccines. Host-derived adjuvants such as CCL28, CCL27, RANTES, TCA3, and GM-CSF have shown significant improvements in immune responses, highlighting their potential to bolster both systemic and mucosal immunity. This underscores the importance of host-derived adjuvants in vaccine development and their advantages over traditional synthetic options. In addition, while these adjuvants offer numerous advantages, including improved compatibility, precise immune activation, and the ability to mimic natural immune responses, the study emphasizes that diagnostic biomarker molecules may not be suitable as adjuvants due to their proinflammatory activity during MERS-CoV infection.

As research progresses, the integration of host-derived adjuvants into vaccine formulations could lead to safer and more effective immunization strategies, ultimately enhancing protection against infectious diseases. Future studies should prioritize optimizing the delivery and combination of these adjuvants to maximize their immunological benefits, paving the way for innovative vaccine development.

## Future perspectives

A structured framework has been implemented to categorize biomarkers by molecular type, function, and supporting evidence (**Table 5**), providing a clear hierarchy for MERS-CoV therapeutic development. Molecules are classified into diagnostic, therapeutic, and immunomodulatory roles, while host-derived adjuvants are grouped based on functional properties such as chemokines and cytokines.

**Table 5:** Host-derived adjuvants for vaccine development.

Category	Molecule	Function	Rationale as a Target	Available/In-Development Products	Level of evidence	Ref
Chemokines	CXCL10/IP-10	Angiogenic chemoattractant, enhances immune recruitment	Adjuvant: Highly expressed in response to viral infections; potential for enhancing vaccine efficacy	Not specific to MERS; tested in other viral models	Strong	(136, 139)
	CX3CL1	Immune function, enhances leukocyte adhesion	Therapy: Multifunctional role in immunity and homeostasis	Not known for MERS-CoV	Moderate	(136, 139)
	CCL28	Mucosal immunity, T-cell recruitment	Adjuvant: Potential role in enhancing mucosal vaccine responses	Some experimental studies in flu vaccines	Moderate	(140, 141)
	CCL2/MCP-1	Regulating monocyte infiltration.	Diagnosis: Linked to severe disease; blockade could mitigate inflammation	Some experimental studies in renal disease	Moderate	(194)
	CXCL8/IL-8	Involved in neutrophil recruitment	Diagnosis: Linked to severe disease; blockade could mitigate inflammation	Anti- CXCL8 (preclinical data in viral infections)	Moderate	(33)
	CCL5/RANTES	Involved in monocyte and T-cell recruitment	Diagnosis: Linked to severe disease; blockade could mitigate inflammation	Met-RANTES (Have been tested *in vivo* against RSV)	Moderate	(38)
	CCL3	Involved in monocyte and T-cell recruitment	Adjuvant: Improved systemic and mucosal immunity	Some preclinical data in viral infections and cancer	Moderate	(136, 139)
	CCL7	Chemoattractant for leukocytes, and activated T lymphocytes	Adjuvant: Improved systemic and mucosal immunity	Some preclinical used in cancer	Moderate	(139)
	CCL27	Mucosal immunity, T-cell recruitment	Adjuvant: Improved systemic and mucosal immunity	preclinical data in SARS-CoV-2	Moderate	(140–143)
	CCL19	Involved in T cell recruitment	Adjuvant: Potent inducer of T cell proliferation	Some preclinical data in viral infections and cancer	Moderate	(136, 139)
	CCL20	Attraction of immune cells including DC, T and B-lymphocytes	Adjuvant: Improved systemic and mucosal immunity	Some preclinical data in viral infections	Moderate	(195)
	CCL21	Involved in T cell recruitment	Adjuvant: Potent inducer of T cell proliferation	Some preclinical data in viral infections and cancer	Moderate	(196)
	XCL1	Attracting T cell and NK cell	Adjuvant: Enhanced effects of CTL and NK cell activation and increased production of IL-2 and INF-γ	Some experimental studies in flu vaccines	Moderate	(136, 139)
	CXCL12	Migration and activation of hematopoietic progenitor cells, endothelial cells, and leukocytes	Adjuvant: Improved systemic and mucosal immunity	preclinical adjuvants used in cancer vaccines	Moderate	(197)
Cytokines	GM-CSF	Pro-inflammatory, enhances antigen presentation	Adjuvant: Enhances vaccine-induced immune responses	GM-CSF adjuvants used in cancer vaccines	Moderate	(168)
	IL-6	Pro-inflammatory cytokine	Diagnosis: Linked to severe disease; blockade could mitigate inflammation	Tocilizumab (approved for inflammatory diseases)	Strong	(60, 61)
Defensins	Human β-defensin 2	Antimicrobial, immune modulation	Adjuvant: Enhances vaccine responses, antiviral properties	Some preclinical data in viral infections	Strong	(122)
Co-stimulatory Molecules	CD40L	Enhances antigen-presenting cell activation	Adjuvant: Improves adaptive immune responses	CD40 agonists in cancer immunotherapy	Strong	(130)
Human antimicrobial	LL-37	Modulate the activities of various immune cells, including dendritic cells	Adjuvant: Induces mucosal and systemic immune responses.	preclinical data in MERS-CoV infections	Moderate	(135)

Host-derived adjuvants is an area with much potential impact on vaccine development. The identified high-priority therapeutic targets including CXCL10/IP10 and IL-6 warrant monoclonal antibody development and clinical trials to reduce immunopathology and improve clinical outcomes. Chemokines such as CCL5, CCL27 and CXCL8 can be used as prognostic biomarkers. High-priority adjuvants such as CD40L, CXCL1, HBD-2, LL-37 and GM-CSF have higher criteria as immune adjuvants which can be a precise implementation of clinical trials. HBD-2 possesses multiple functions involved in determining innate and adaptive immunity: it has a direct antimicrobial function and can act against a broad range of pathogens by disrupting membrane integrity, acts as a chemotactic factor for neutrophils and T lymphocytes, promotes the maturation of dendritic cells for enhancing the presentation of antigens, modulates signaling pathways and inflammatory response, and also stimulates the production of pro-inflammatory cytokines for amplifying immune responses. On the other hand, CD40L (CD154) is a co-stimulatory protein expressed on activated T cells, and its interaction with CD40 receptors on antigen-presenting cells (APCs) stimulates them and increases their ability to present antigens. This interaction induces B cell proliferation and antibody production, dendritic cell maturation and secretions of cytokines IL-12, which is essential for T helper cell differentiation. Taken together, the unique mechanisms of HBD-2 and CD40L make them useful and excellent candidates as adjuvants in the design of safer and more effective MERS-CoV vaccine. Future studies should focus on clinical trials with adjuvants of human origin, and exploration of new biomarkers of disease progression that may help to elucidate the precise mechanisms of MERS-CoV immunity which can inform the rational development of vaccines utilizing human-derived adjuvants. These studies should focus on their ability to enhance both systemic and mucosal immunity.
